# Designing Behavioral Feedback Visualizations to Support Health Behavior Change

**DOI:** 10.1093/geroni/igab046.477

**Published:** 2021-12-17

**Authors:** Qiong Nie, Daniel Morrow, Maurita Harris, Wendy Rogers

**Affiliations:** 1 University of Illinois at Urbana Champaign, Champaign, Illinois, United States; 2 University of Illinois Urbana-Champaign, Champaign, Illinois, United States

## Abstract

Health technology has the potential to support behavior change by measuring performance and providing users with visualizations of this performance as feedback. Such visual feedback has had limited success in changing health behaviors, but it is not clear why. We conducted a systematic review of the visual feedback literature to develop an organizational framework representing the visual feedback-action process. We identified the components that have been investigated in the context of visual feedback. These components are classified into four categories: visualization types (e.g., bar graph) and variables (e.g., color); feedback characteristics (e.g., social comparison); psychological processes (e.g., motivation) and action (e.g., exercise). The insights will inform the design of feedback visualizations in a smartphone application to support medication adherence for older adults. More broadly, this integrative perspective will yield principles of feedback visualization techniques and components that influence the behavior change process and develop a roadmap to facilitate the design.

